# Comparative neuronal differentiation of self-renewing neural progenitor cell lines obtained from human induced pluripotent stem cells

**DOI:** 10.3389/fncel.2013.00175

**Published:** 2013-10-07

**Authors:** Chiara Verpelli, Luigi Carlessi, Giulia Bechi, Elena Fusar Poli, Daniel Orellana, Christopher Heise, Silvana Franceschetti, Renato Mantegazza, Massimo Mantegazza, Domenico Delia, Carlo Sala

**Affiliations:** ^1^CNR Institute of Neuroscience and Department of Biotechnology and Translational Medicine, University of MilanMilan, Italy; ^2^Department of Experimental Oncology, Fondazione IRCCS Istituto Nazionale dei TumoriMilan, Italy; ^3^Department of Neurophysiopathology, Foundation Carlo Besta Neurological InstituteMilan, Italy; ^4^Neuromuscular Diseases and Neuroimmunology, Foundation Carlo Besta Neurological InstituteMilan, Italy; ^5^Institute of Molecular and Cellular Pharmacology, LabEx ICST, CNRS UMR7275 and University of Nice-Sophia AntipolisValbonne, France

**Keywords:** induced pluripotent stem cells, neuronal differentiation, synapse formation, Human neurons, postsynapse

## Abstract

Most human neuronal disorders are associated with genetic alterations that cause defects in neuronal development and induce precocious neurodegeneration. In order to fully characterize the molecular mechanisms underlying the onset of these devastating diseases, it is important to establish *in vitro* models able to recapitulate the human pathology as closely as possible. Here we compared three different differentiation protocols for obtaining functional neurons from human induced pluripotent stem cells (hiPSCs): human neural progenitors (hNPs) obtained from hiPSCs were differentiated by co-culturing them with rat primary neurons, glial cells or simply by culturing them on matrigel in neuronal differentiation medium, and the differentiation level was compared using immunofluorescence, biochemical and electrophysiological methods. We show that the differentiated neurons displayed distinct maturation properties depending on the protocol used and the faster morphological and functional maturation was obtained when hNPs were co-cultured with rat primary neurons.

## Introduction

Neuronal disorders in humans can be caused by defects in neuronal development and neurodegenerative processes, and are often related to functional alterations of cortical neurons (Calahorro and Ruiz-Rubio, 2011; Penzes et al., 2011; Esposito et al., 2012; Marcello et al., 2012; Verpelli and Sala, 2012). In order to fully characterize the molecular mechanisms underlying the onset of these diseases, it is important to establish new *in vitro* models able to recapitulate the human pathology as closely as possible.

The recently developed techniques which allow the neural differentiation of skin fibroblast-derived human induced pluripotent stem cells (hiPSCs) represent an innovative strategy to study neuronal development and degeneration, circuit formation and function, and for generating new *in vitro* human models of brain diseases (Takahashi and Yamanaka, 2006; Dimos et al., 2008; Wernig et al., 2008; Marchetto et al., 2010; Xu et al., 2010; Ricciardi et al., 2012; Yamanaka, 2012; Carlessi et al., 2013a). For this reason, it is crucial to define a reproducible method for obtaining wild type and disease-derived mature neurons which can be employed for functional and comparative analysis. Previous works have described several methods to obtain self-renewing neural progenitors (hNPs) from embryonic stem cells (ESCs) (Reubinoff et al., 2001; Zhang et al., 2001; Peng and Chen, 2005; Axell et al., 2009) and from hiPSCs (Wernig et al., 2008; Shi et al., 2012; Carlessi et al., 2013b), which can then be successfully differentiated into patient-derived neurons (Marchetto et al., 2010; Zhang et al., 2010; Pasca et al., 2011; Aboud et al., 2012; D'aiuto et al., 2012; Farra et al., 2012; Israel et al., 2012; Paulsen et al., 2012; Xia et al., 2012; Yin et al., 2012; Salewski et al., 2013). As various protocols have been employed for this purpose, a comparative study describing the timing and level of differentiation achieved with different methodologies could help decide the best suitable approach to obtain neurons to be used as *in vitro* models of neurological diseases.

Here we followed a reproducible technique for obtaining hNPs from hiPSCs, which we then differentiated into neurons applying three different protocols: co-culture with rat primary neurons or glial cells or culture on matrigel, showing that they display distinct differentiation properties depending on the protocol used.

## Materials and methods

### Cell culture and hiPSC generation

Fibroblasts were obtained from skin biopsies of three healthy donors (called D1-3) under the approval of the Ethics Board. Cells were maintained and expanded in Dulbecco's modified Eagle medium supplemented with 20% fetal bovine serum and penicillin/streptomycin (P/S) (all from *Invitrogen*). Fibroblasts were infected with STEMCCA Cre-Excisable Constitutive Polycistronic Lentivirus (Millipore) following the manufacturer's instructions. After 25 days, hiPSC clones were manually picked and directly transferred on mitomycin C-treated mouse embryonic fibroblasts in human ESC medium composed of DMEM/F12 containing 20% KSR (vol/vol), 10 ng ml^−1^ bFGF, 1 mM glutamine, 100 μm non-essential amino acids, 100 μ M 2-mercaptoethanol, 50 U ml^−1^ penicillin and 50 mg ml^−1^ streptomycin (all from *Invitrogen*).

### Generation of neural progenitor cells

At least one clone for each donor was processed for neural differentiation, but the results of the experiments were obtained with clones #1 of donors 1 and 2 (D1 and D2), and both clones produced exactly the same results.

hiPSC lines were detached with Collagenase IV and resuspended in human ESC medium without bFGF to form embryoid bodies (EBs), which were cultured in suspension in low adhesion non-treated sterile dishes (Nunc). After 5 days, EBs were collected and plated in matrigel-coated dishes and grown for additional 4 days in 1X N2 media supplemented with 20 ng/ml bFGF in order to obtain neural rosettes. Rosettes were then manually picked, resuspended to single cells in NP medium, composed of DMEM/F12 containing 2 mM glutamax, B27 1:500, N2 1:100, 1% P/S, 20 ng/ml EGF, 20 ng/ml bFGF, and plated in matrigel-coated flasks. NP medium was changed every 2 days; once the cell culture reached 95% confluence, cells were dissociated with Accutase (*Invitrogen*). After dissociation with Accutase 1 × 10^7^ cells were blocked in 1% BSA in PBS and incubated with 20 μ L of anti-PSA-NCAM antibody conjugated with magnetic micro-beads (Cat. No. 130-092-966, Miltenyi Biotec) for 15 min at 4°C. After washing, the cell suspension was loaded on the separation column and collected through the magnet provided by the Miltenyi Biotec company. Negatively-labeled cells which passed through during column-washing were discarded, whereas positively-labeled cells that remained in the column were eluted to another tube with culture media after removing the column from the magnetic stand. These were further cultured and expanded up to 30 passages (Kim et al., 2012).

### Terminal differentiation of hNPs and lentivairal infection

hNPs were differentiated following different protocols and the experiments were repeated on two lines of hNPs (obtained from hiPSC clone #1 of D1 and hiPSC clone #1 of D2) at the same passages (at passage 5 or 6). For differentiation on rat cortical neuronal cells, primary rat cultures were extracted from 18- to 19-day-old rat embryos (pregnant female rats were obtained from Charles River Laboratories). The neurons were plated at medium density (150–200 cells/mm^2^) and grown as described in Verpelli et al. (2010); at DIV 7, 20,000 hNPs infected with Syn:EGFP were plated onto the cortical neurons; medium was changed every 4 days until day 88.

For differentiation on glial cells, rat glia was prepared as described in Goslin and Banker (1991) and 20,000 hNPs were plated onto 75,000 glial cells. Two days after plating, glial medium was replaced with Neurobasal medium supplemented with B27 (*Invitrogen*); medium was changed every 4 days until day 63.

For differentiation with medium, 4000 hNPs were detached and plated on matrigel-coated coverslips in NP medium without bFGF and EGF. Medium was changed every 4 days until day 63.

### Immunocytochemistry

Cells were fixed in 4% paraformaldehyde and 4% sucrose at room temperature or in 100% methanol at −20°C. Primary and secondary antibodies were applied in GDB buffer composed of 30 mM phosphate buffer, pH 7.4, containing 0.2% gelatin, 0.5% Triton X-100, and 0.8 mM NaCl. Primary antibodies were applied for 3 h at room temperature or overnight at 4°C. Secondary antibodies were applied for 1 h at room temperature.

Confocal images of 1024 × 1024 pixels were obtained with a LSM 510 Meta confocal microscope (Carl Zeiss, a gift from Fondazione Monzino) and a 63× objective with sequential acquisition settings. Each image was a Z-series projection of 7–15 images, each averaged 2–4 times, and taken at 0.4–0.7-μ m depth intervals.

### Antibodies

The following antibodies were used: rabbit anti-NANOG (Abcam), mouse anti-OCT3\ 4 (Santa Cruz Biotechnology), mouse anti-TRA-1-81 (eBioscience), mouse anti-SSEA4 (eBioscience), mouse anti-β Tubulin III (Sigma), mouse anti-SOX17 (R&D), mouse aSMA (Sigma), mouse anti-vinculin (Sigma), mouse anti-Nestin (Chemicon), mouse anti-Neuronal Nuclei (Millipore), mouse anti-MAP2 (Abcam), mouse anti-Human Nuclei (Millipore), rabbit anti-VGLUT (Synaptic System), mouse anti-synaptophysin (Sigma), mouse and rabbit anti-GFAP (Sigma), mouse anti-PSD-95 (NeuroMab, University of California, Davis/NIH NeuroMab Facility); rabbit anti-VGAT (Synaptic System), mouse anti-SCG10 (NeuroMab), mouse anti-Pan-KChIP (NeuroMab), rabbit anti-GABA (Sigma) and mouse anti-GAD67 (Millipore), rabbit anti-PAX6 (Covance), rabbit anti-Ki67 (Thermo Scientific), mouse anti-Sox2 (Abcam); secondary FITC-, Cy3- and Cy5-conjugated anti-mouse and anti-rabbit (Jackson ImmunoResearch); secondary HRP-conjugated anti-mouse and anti-rabbit (GE Healthcare).

### Electrophysiological recordings and analysis

When plated on primary cortical neurons or on primary glial cells, hNP-derived neurons were selected by their fluorescence. Recordings were done at room temperature (22–25°C) using a Multiclamp 700A patch-clamp amplifier and pClamp 10.2 software (Molecular Devices) as in Cestèle et al. (2008). For the recordings of total ionic currents, signals were filtered at 10 kHz and sampled at 100 kHz. We recorded sodium currents using the whole-cell configuration of the patch-clamp technique. Recordings were usually started 5 min after the rupture of the membrane patch, to allow intracellular dialysis with the pipette solution. The external bath solution contained the following (in mM): 129 NaCl, 1.25 NaH_2_PO_4_, 35 glucose, 1.8 MgSO_2_, 1.6 CaCl_2_, 3 KCl and 10 HEPES, pH 7.4 with NaOH; the internal pipette solution contained the following (in mM): 120 K-gluconate, 15 KCl, 2 MgCl_2_, 0.2 EGTA, 10 HEPES, 20 P-creatine, 2 Na_2_ATP, 0.2 Na_2_GTP and 0.1 Leupeptine, pH 7.2 with KOH. Cell capacitance and series resistance errors were carefully compensated (~85%) throughout the experiment. Pipette resistance was between 2.6 and 3.0 MΩ. For the recordings of postsynaptic currents, signals were filtered at 3 kHz and sampled at 10 kHz. We used 3 mM kynurenic acid and 10 μ M bicuculline to block glutamatergic and GABAergic postsynaptic currents, respectively. Postsynaptic currents were identified as the events larger than 2 times the RMS noise of the eventless periods in the trace. When we switched to current-clamp mode, we applied the bridge balance compensation and recorded neuronal firing by injecting depolarizing current pulses of increasing amplitude; for these experiments, we held the resting potential at −70 mV by injecting the appropriate holding current, in order to compare the firing of different cells in the same conditions. The neurons with unstable resting potential and/or unstable firing were discarded. In current clamp mode, signals were filtered at 10 kHz and sampled at 20 kHz.

### Western blotting

Total cell extracts and Western blot analysis were performed as previously described in Carlessi et al. (2009). Briefly, cells were lysed in Laemmli buffer (0.125 M Tris–HCl pH 6.8, 5% SDS) and lysates were sonicated, size-fractionated by SDS–PAGE and electroblotted onto PVDF membranes (Millipore, Bedford, MA), which were incubated with the indicated primary antibodies. Binding of the antibodies to the membranes was detected using peroxidase-conjugated secondary antibodies and ECL (Pierce, Rockford, IL) on autoradiographic films. Bands were acquired with a digital scanner.

### RNA isolation and PCR analysis

All RNA samples were extracted with RNeasy Micro Kit (Quiagen) from about 1 to 5 × 10^6^ cells, according to the manufacturer's instructions. RNAs were retro-transcribed using the Transcriptor High Fidelity cDNA Synthesis Kit (Roche) and cDNAs were amplified by Amplibiotherm DNA polymerase (Fisher Molecular Biology). For qRTPCR, we used the STEMCCA Viral Gene Detection qPCR Multiplex Kit (Millipore).

### Virus construct and production

Lentiviruses were produced in 293T packaging cells as previously described in Naldini et al. (1996) and Lois et al. (2002).

## Results

### Generation of hiPSC lines

**Figure 1:**
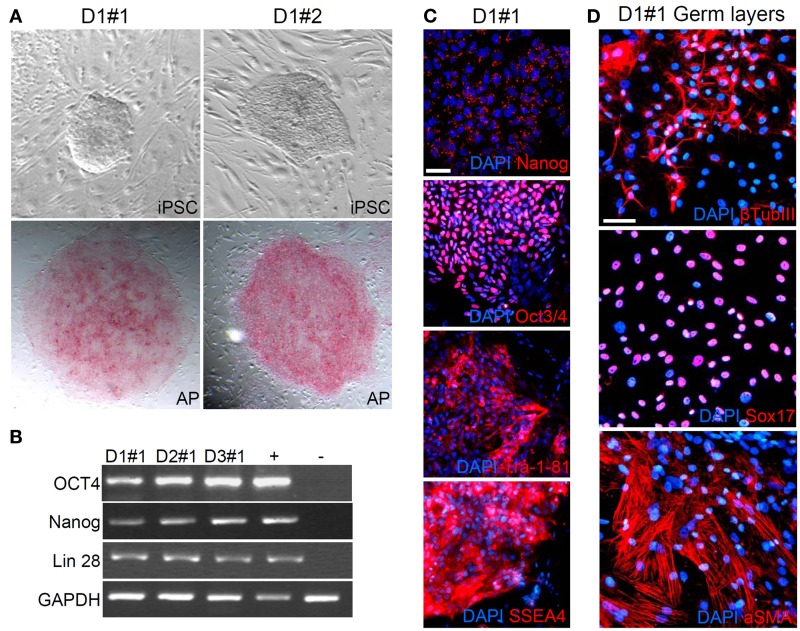
**Generation of hiPSC colonies. (A)** Representative images of established hiPSC colonies, clones are positive for phosphatase alkaline. **(B)** and **(C)** iPSC clones show expression of pluripotency markers. **(D)** iPSCs differentiate into the three germ layers *in vitro*, ectoderm (+β Tubulin III), endoderm (+Sox17) and mesoderm (+aSMA). Scale bar, 50 μm.

**Figure A1:**
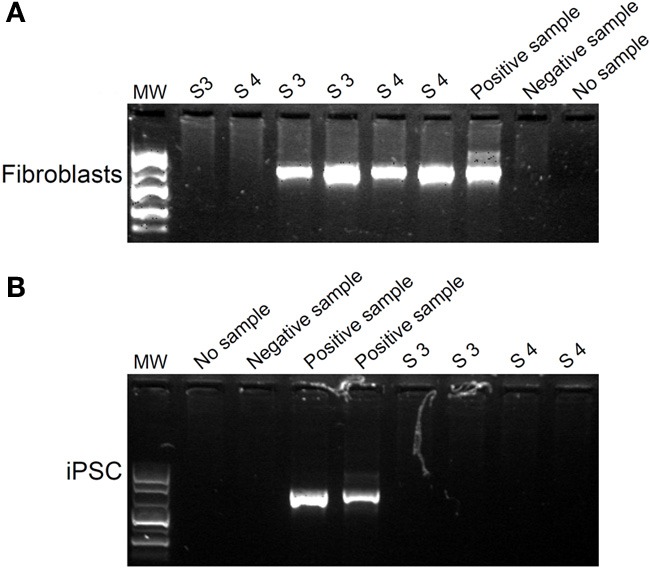
**Treatment of mycoplasma-infected fibroblasts with BM-Cyclin.** The fibroblast culture medium from fibroblasts**(A)** and iPSCs **(B)** was analyzed for the presence of mycoplasma DNA using PCR before **(A)** and after **(B)** treatment for 20 days with BM-Cyclin.

We generated hiPSC lines from fibroblasts obtained from three healthy control donors (called D1-3) using the hSTEMCCA-loxP virus (Millipore) as described in the manufacturer's protocol. We obtained at least 10 independent clones from each fibroblast culture. We maintained the colonies, expressing alkaline phosphatase, on mitomycin-inactivated mouse embryonic fibroblasts (MEFs) (Figure 1A shows two iPS clones, #1 and #2, from donor 1) and the plurypotency of each clone was confirmed by RT analysis of markers such as OCT4, Nanog and Lin28 (Figure 1B, results obtained only from clones #1 of each donor are shown as representative examples). Moreover, all clones also expressed Nanog, Oct 3/4, Tra-1-81 and SSEA4, which were detected by immunofluorescence (Figure 1C, clone #1 from donor 1 is shown as a representative example). We analyzed the ability of our hiPSC clones to spontaneously differentiate into the three germ layers in the absence of mitogenic factors by observing the expression of β Tubulin III, Sox17, and aSMA as markers of ectoderm, endoderm and mesoderm, respectively (Figure 1D, clone #1 from donor 1 is shown as a representative example). Finally, we tested the ability of two independent clones, for each starting fibroblast line (D1-3), to induce teratoma in mice (data not shown) as described in Ricciardi et al. (2012). Interestingly, two of the fibroblast cultures that we reprogrammed were originally infected by mycoplasma and were rescued by extensive use of BM-Cyclin (ROCHE) before infection with hSTEMCA-loxP virus. The hiPSC colonies we obtained were completely free of mycoplasma (Figures A1A,B).

### Generation and characterization of human self-renewing neural progenitors (hNPs)

**Figure 2:**
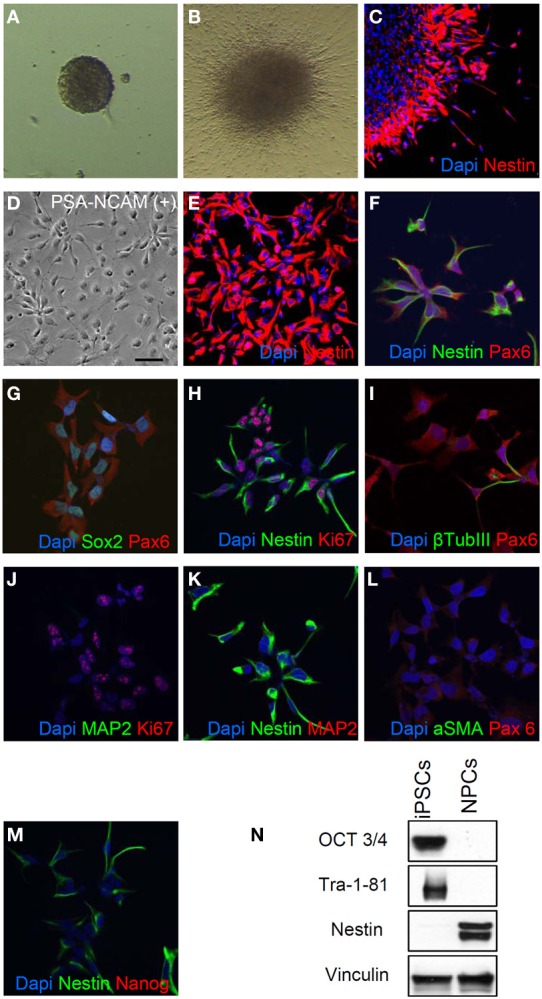
**Generation of neural progenitor cells (hNPs). (A,B)** Representative images of embryoid bodies **(A)** and rosettes **(B)**. **(C)** Rosettes are positive for the early neural precursor marker Nestin. **(D)** This representative image shows the typical morphology of PSA-NCAM positive purified hNPs, **(E–H)** Most of PSA-NCAM positive cells are positive for Nestin, Pax6 and Sox2, typical hNP makers; they are also positive for Ki67 confirming that they are self-renewing. **(I)** about 5% of cells are positive for β Tubulin III. **(J–L)** hNPs do not express MAP2 and αSMA, markers of mature neurons and mesodermal cells respectively. **(M,N)** hNPs do not express the pluripotency markers Nanog, OCT3/4 and Tra-1-81. Scale bar, 50 μm.

We started neural differentiation by inducing the formation of embryoid bodies (EBs) from hiPSC colonies at passage 5 (Figure 2A). We then plated the embryoid bodies onto matrigel-coated dishes and grew them in a medium supplemented with N2. After 5–7 days in culture, attached EBs differentiated into rosettes (Figure 2B) which expressed the early neural precursor marker Nestin (Figure 2C). Rosettes were then dissociated with Accutase or manually picked, plated in matrigel-coated dishes and maintained as neural progenitors using a medium containing N2, B27, bFGF, and EGF. We purified a homogeneous population of hNPs using anti-PSA-NCAM microbeads and magnetic-based separation (Kim et al., 2012) (Figure 2D).

All purified hNPs were positive for the neural cell markers Nestin, Pax6 and Sox2 (Figures 2E–H) and the majority of the cells were positive for Ki67, strongly demonstrating that they are self-renewing (Figure 2F); β TubIII positive cells were present, in accordance with previous reports (Elkabetz and Studer, 2008; Kim et al., 2012) (Figure 2I), and we didn't detect positivity for the neuron-specific protein MAP2 (Figures 2J,K) and the myoepithelial marker αSMA (Figure 2L). In addition, hNPs did not express the pluripotency markers Nanog OCT3/4 and Tra-1-81 (Figures 2M,N).

**Figure A2:**
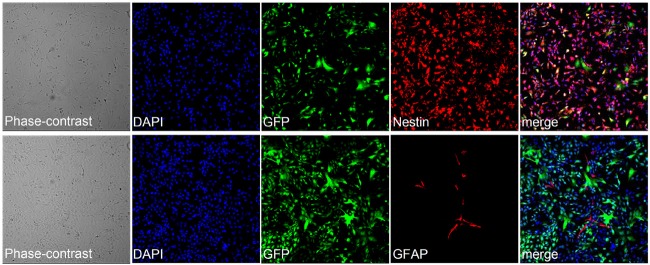
**Stem cell features of hNPs after multiple passages.** After 16 passages, hNPs are still positive for Nestin (top panel). A low number of cells are positive for GFAP (bottom panel). The cells do not spontaneously differentiate during the multiple passages and maintain their stem cell nature.

hNPs were kept under proliferating conditions in the presence of bFGF and EGF and were stable in morphology and for the expression of Nestin for more than 30 passages (data not shown). Moreover, hNPs could be successfully infected with lentiviruses expressing different proteins. We thus obtained different hNP clones expressing EGFP under CMV and Synapsin promoters (see Figure 4, SYN-GFP), RFP under CMV promoter, PSD-95-GFP (not shown) and GFP-Homer1b under CMV promoters (see Figure 8, HOMER-GFP). All clones were extensively expanded, frozen and thawed without losing their ability to further differentiate into neurons up to passage 16. The large majority of the cells were positively stained for Nestin and only a very small number of them were also positive for the astroglial marker GFAP (Figure A2).

### Terminal neuronal differentiation of hNPs

To differentiate hNPs into functional neurons, we compared three different protocols: we cultured the hNPs on E18 rat primary cortical neurons (first method), on rat primary glial cells (second method) or simply on matrigel (third method). In order to characterize the best and fastest method to induce hNP differentiation, each condition was analyzed and compared for morphology and electrophysiological properties at 20, 50, and 60 days after plating.

For the culture on E18 rat primary cortical neurons, hNPs were first infected with a lentivirus expressing EGFP under either the CMV (EGFP-hNPs) or Synapsin promoter, then plated onto cortical neurons at DIV7 (10,000–60,000 infected hNPs were plated in one well of a twelve well plate containing 75,000 primary neurons). Neuronal medium (Romorini et al., 2004) was changed every 4 days until the end of differentiation.

The hNP-derived cells could be distinguished from rat neurons based on GFP expression or by labeling them with human-specific antibodies. When infected with EGFP under the Synapsin promoter, neurons expressed GFP after 30 days in culture.

**Figure 3:**
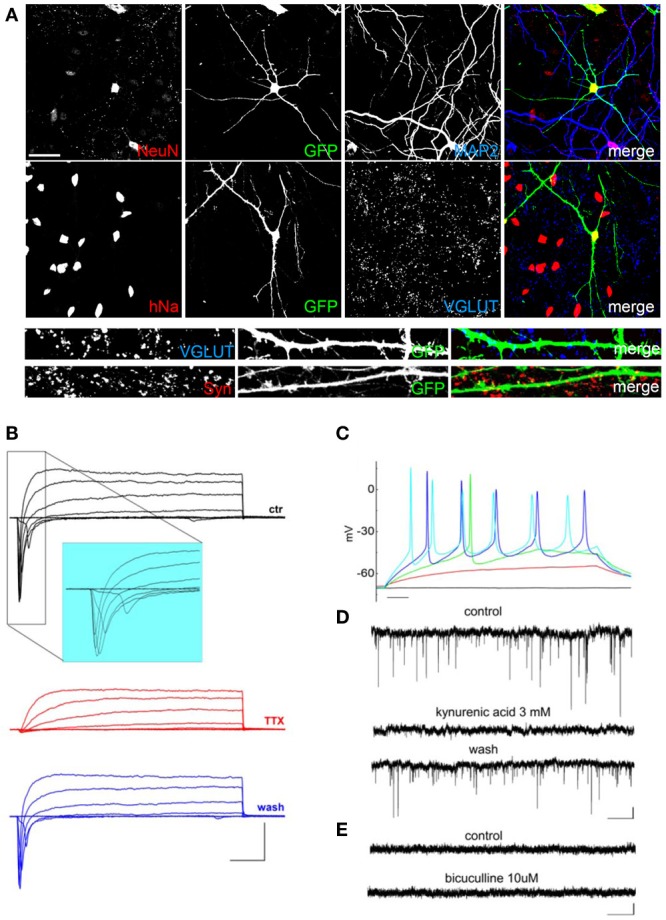
**Characterization of hNP-derived neurons differentiated by coculture on E18 rat primary cortical neurons. (A)** hNP-derived neurons are positive for MAP2 and hNA and are also positive for the mature neuronal markers NeuN (red, top, scale bar 50 μm), VGLUT (blue), and synaptophysin (red, bottom, scale bar 15 μm). **(B)** Representative total ionic current traces recorded with depolarizing voltage steps between-70 and + 10 mV (10 mV increments from a holding potential of −70 mV) from hNP-derived neurons. From top to bottom: currents in control (black), with perfusion of tetrodotoxin 1 μ M (red) and after washout (blue). Scale bars, 10 ms, 1000 pA. The inset shows an enlargement of the traces in control, for better displaying voltage gated sodium currents. **(C)** Representative firing traces recorded from hNP-derived neurons during application of 1 s injections of depolarizing current steps from a holding potential of −70 mV. Scale bar, 100 ms. **(D)** Traces showing spontaneous excitatory postsynaptic currents (EPSCs) recorded from hNP-derived neurons. From top to bottom: EPSCs recorded at the holding potential of −70 mV in the presence of 10 μ M bicuculline, during perfusion of bicuculline and 3 mM kynurenic acid (which blocked the activity), and after washout of kynurenic acid. Scale bars, 10 pA, 1 s. **(E)** Current traces acquired at the holding potential of +30 mV with 3 mM kynurenic acid for recording spontaneous inhibitory (GABAergic) postsynaptic currents (IPSCs), which we did not observe. From top to bottom: current traces before and after application of 10 μ M bicuculline. Scale bars, 10 pA, 1 s.

After 20–60 days of differentiation, we evaluated the hNP-derived mature neuronal cells using specific markers visualized by immunofluorescence. The hNP-derived neurons, displaying green fluorescence, all expressed nuclear NeuN (Figure 3A), a classical marker of neuronal cell types; interestingly, immunoreactivity for NeuN is only observed in neurons that have become postmitotic, whereas no staining is observed in proliferative zones (Mullen et al., 1992). All green cells also expressed the human nuclei protein (hNa), demonstrating that the neuronal cells we obtained derived from human cells (Figure 3A). As the infection efficiency of the EGFP-expressing lentivirus was lower than 100%, some non-green cells were also positive for hNa. We analyzed neuronal differentiation by immunofluorescence using MAP2 for labeling dendrites and VGLUT and Synaptophysin (Syn) for labeling synapses. hNP-derived neurons had elaborate dendritic arbors and many Synaptophysin-positive puncta, and VGLUT positive puncta were detected starting around day 60 of differentiation (Figure 3A bottom). We observed that the majority of the neurons generated from hNP cell cultures were MAP2 and VGLUT positive neurons, and we basically did not detect MAP2 and GABA double-positive cells (data not shown), indicating that we mainly obtained excitatory neurons.

We recorded total ionic currents, discharges induced by injection of depolarizing currents, and spontaneous postsynaptic currents. Recordings of total ionic currents showed that hNPs cultured on E18 rat primary cortical neurons were able, after 60 days in culture, to generate cells displaying voltage-gated potassium currents and, most importantly, voltage-gated sodium currents blocked by the application of the selective blocker tetrodotoxin (TTX), which is a specific feature of mature neurons (Figure 3B). These cells were excitable and displayed discharges of action potentials lasting for the entire duration of the 400 ms step of injected depolarizing current (Figure 3C). In order to determine whether hNP-derived neurons expressed functional neurotransmitter receptors and formed functional synapses, we recorded spontaneous glutamatergic and GABAergic postsynaptic currents (excitatory postsynaptic currents, EPSCs and inhibitory postsynaptic currents, IPSCs). EPSC were recorded at a potential of −70 mV, IPSCs at a potential of +30 mV. We were able to record EPSCs that were blocked by application of kynurenic acid (Figure 3D); on the contrary, we have never observed GABAergic postsynaptic currents in these conditions (Figure 3E).

**Figure 4:**
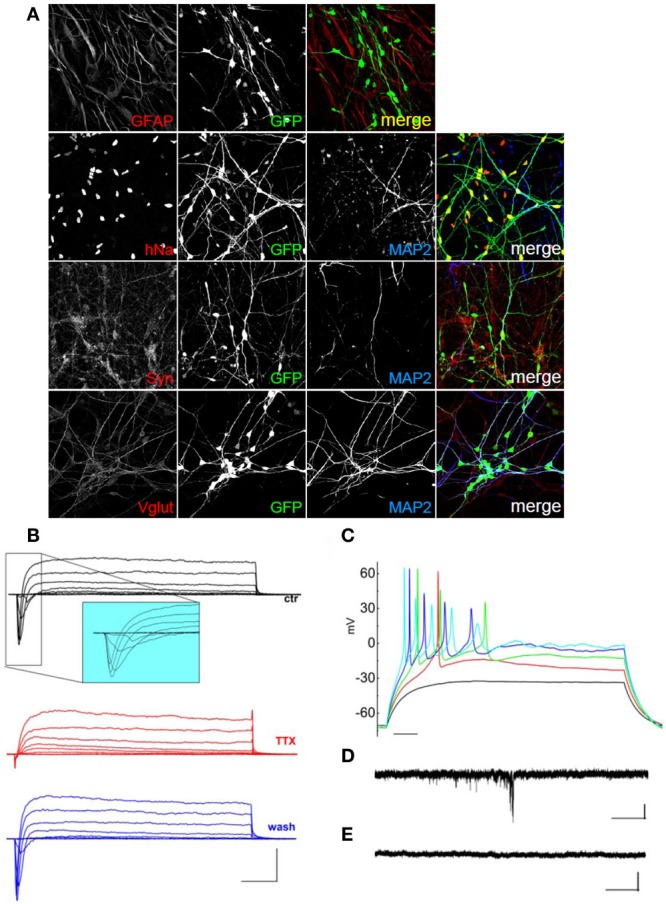
**Characterization of hNP-derived neurons differentiated by co-culture on rat glial cells. (A)** hNP-derived neurons are positive for hNA (red; top, left) and MAP2 (blue) and for the mature neuronal markers synaptophysin (red, middle, left) and VGLUT (red, bottom, left). Scale bar, 50 μm. **(B)** Representative total ionic current traces recorded with depolarizing voltage steps between −70 and +10 mV (10 mV increments from a holding potential of −70 mV) from hNP-derived neurons. From top to bottom: currents in control (black), with perfusion of tetrodotoxin 1 μ M (red) and after washout (blue). Scale bars, 10 ms, 500 pA. The inset shows an enlargement of the traces in control, for better displaying voltage gated sodium currents. **(C)** Representative firing traces recorded from hNP-derived neurons during application of 1 s injections of depolarizing current steps from a holding potential of −70 mV. Scale bar, 100 ms. **(D)** Traces showing spontaneous excitatory postsynaptic currents (EPSCs) recorded from hNP-derived neurons. EPSCs recorded at the holding potential of −70 mV in the presence of 10 μ M bicuculline. Scale bars, 10 pA, 1 s. **(E)** Current traces acquired at the holding potential of + 30mV with 3 mM kynurenic acid for recording spontaneous inhibitory (GABAergic) postsynaptic currents (IPSCs), which we did not observe. Scale bars, 10 pA, 1 s.

We next investigated whether we could differentiate hNPs using rat primary glial cells as feeders. hNPs were first infected with a lentivirus expressing EGFP under the Synapsin promoter, then 10,000–40,000 infected hNPs were plated in one well of a twelve well plate containing 75,000 glial cells. When infected with EGFP under the Synapsin promoter, neurons expressed GFP after 30 days in culture. After 60 days differentiated cells, green, were positive for hNa, but also for neuronal and synaptic markers such as MAP2, synaptophysin and VGLUT (Figure 4A).

Again we recorded total ionic currents, discharges induced by injection of depolarizing current, and spontaneous postsynaptic currents. hNPs, cultured on primary glial cells, were able to generate neuronal cells displaying voltage-gated potassium currents and, most importantly, voltage-gated sodium currents blocked by the application of TTX (Figures 4B,C).

**Figure 5:**
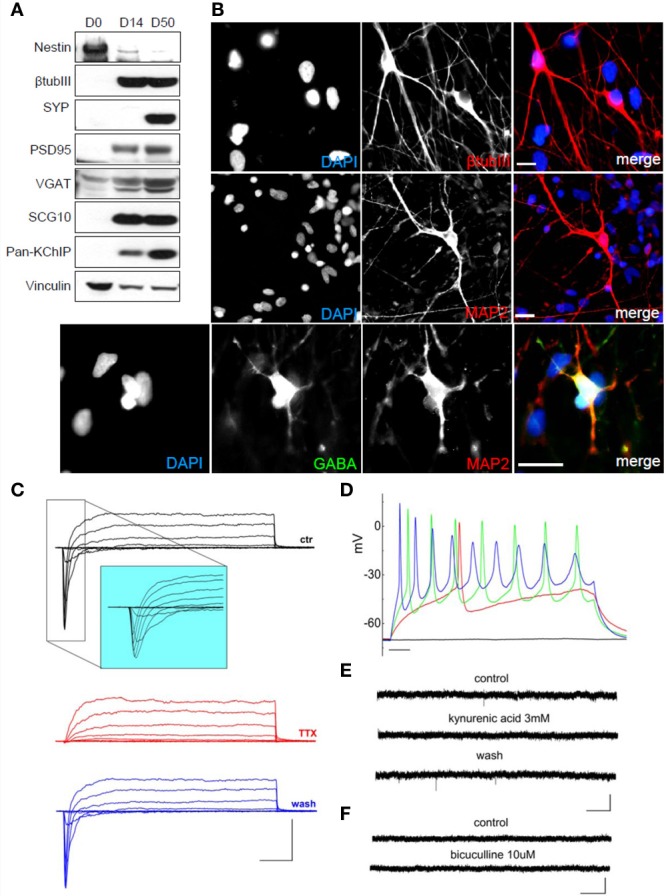
**Characterization of hNPC-derived neurons grown on matrigel in differentiation medium. (A)** During differentiation of hNPs, the neural precursor marker Nestin is downregulated while the neuronal marker β TubIII is upregulated. hNP-derived neurons express the pre-synaptic marker synaptophysin, the postsynaptic marker PSD-95, the inhibitory synapse marker VGAT, the neuronal growth-associated protein SCG10 and the K^+^ channel interacting proteins KChIP. **(B)** hNP-derived neurons are positive for the neuronal markers β TubIII (red, top), MAP2 (red, middle and bottom) and GABA (green). Scale bar, 20 μm. **(C)** Representative total ionic current traces recorded with depolarizing voltage steps between −70 and + 10 mV (10 mV increments from a holding potential of −70 mV) from hNP-derived neurons. From top to bottom: currents in control (black), with perfusion of tetrodotoxin 1 μ M (red) and after washout (blue). Scale bars, 10 ms, 500 pA. The inset shows an enlargement of the traces in control, for better displaying voltage gated sodium currents. **(D)** Representative firing traces recorded from hNP-derived neurons during application of 1 s injections of depolarizing current steps from a holding potential of −70 mV. Scale bar, 100 ms. **(E)** Traces showing spontaneous excitatory postsynaptic currents (EPSCs) recorded from hNP-derived neurons. From top to bottom: EPSCs recorded at the holding potential of −70mV in the presence of 10 μ M bicuculline, during perfusion of bicuculline and 3 mM kynurenic acid (which blocked the activity), and after washout of kynurenic acid. Scale bars, 10 pA, 1 s. **(F)** Current traces acquired at the holding potential of + 30 mV with 3 mM kynurenic acid for recording spontaneous inhibitory (GABAergic) postsynaptic currents (IPSCs), which we did not observe. From top to bottom: current traces before and after application of 10 μ M bicuculline. Scale bars, 10 pA, 1 s.

Using the voltage-clamp configuration we also recorded spontaneous glutamatergic postsynaptic currents (Figure 4D), but we never observed GABAergic postsynaptic currents (Figure 4E). Finally, we differentiated hNPs into neurons in the absence of feeder cells. 4000 hNPs were plated onto matrigel-coated coverslips and grown in hNP medium without bFGF and EGF for up to 60 days. The medium was changed two times a week. As shown in Figure 5A, total cell extracts were analyzed by western blot at day 0, 14, and 50 of differentiation. At day 14 they were positive for βTubulin III and PSD-95, while the expression of Nestin was reduced, and synaptic development continued until day 50, as shown by the increasing levels of synaptophysin and PSD-95. hNP-derived neurons were also analyzed by immunofluorescence at day 50 and the majority of cells were positive for the neuronal markers βTubulin III and MAP2 (Figure 5B).

Similar to the previous differentiation methods, neurons from these cultures displayed voltage-gated sodium currents blocked by the application of TTX and voltage-gated potassium currents (Figure 5C) and were excitable (Figure 5D). We recorded spontaneous glutamatergic postsynaptic currents (EPSCs) (Figure 5E), but we never observed GABAergic postsynaptic currents in these conditions (Figure 5F) although we found some spare neurons positive for GABA (Figure 5B, bottom panels).

**Figure 6:**
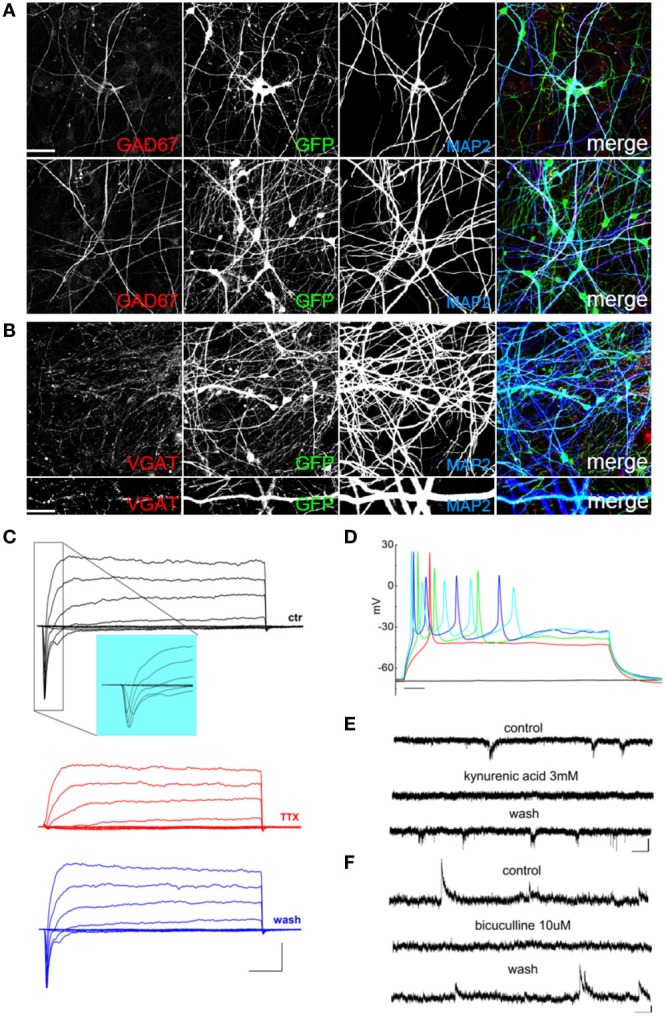
**Retinoic acid-induced differentiation of hNPs into GABAergic neurons. (A)** hNP-derived neurons are positive for MAP2 and for the GABAergic marker GAD67 (red; left). Scale bar, 50 μm. **(B)** hNP-derived neurons are positive for MAP2 and the GABAergic marker VGAT. Scale bar, 15 μm. **(C)** representative total ionic current traces recorded with depolarizing voltage steps between −70 and + 10 mV (10 mV increments from a holding potential of −70 mV) from hNP-derived neurons. From top to bottom: currents in control (black), with perfusion of tetrodotoxin 1 μ M (red) and after washout (blue). Scale bars, 10 ms, 500 pA. The inset shows an enlargement of the traces in control, for better displaying voltage gated sodium currents. **(D)** Representative firing traces recorded from hNP-derived neurons during application of 1 s injections of depolarizing current steps from a holding potential of −70 mV. Scale bar, 100 ms. **(E)** Traces showing spontaneous excitatory postsynaptic currents (EPSCs) recorded from hNP-derived neurons. From top to bottom: EPSCs recorded at the holding potential of−70 mV in the presence of 10 μ M bicuculline, during perfusion of bicuculline and 3 mM kynurenic acid (which blocked the activity), and after washout of kynurenic acid. Scale bars, 10 pA, 1 s. **(F)** Traces showing spontaneous inhibitory (GABAergic) postsynaptic currents (IPSCs) recorded from hNP-derived neurons. From top to bottom: current traces recorded at the holding potential of +30mV in the presence of 3 mM kynurenic acid, during perfusion of kynurenic acid and 10 μ M bicuculline (which blocked the activity), and after washout of bicuculline. Scale bars, 10 pA, 1 s.

Thus, in order to improve the differentiation of GABAergic neurons, we plated EGFP-hNPs onto rat cortical neurons as previously described and treated the coculture with 1μ M retinoic acid starting 24 h after plating (Addae et al., 2012). After 60 days of differentiation, we stained the hNP-derived mature neuronal cells using an anti-GAD67 antibody to detect GABAergic neurons and observed a MAP2, GAD67, and VGAT positive population of cells (Figures 6A,B). In these cultures we recorded voltage-gated sodium and potassium currents (Figure 6C), action potential discharges (Figure 6D), as well as both excitatory and inhibitory postsynaptic currents (Figures 6E,F), showing that retinoic acid promotes the differentiation of GABAergic neurons.

The efficiency of terminal differentiation expressed as percentage of neurons and glial cells among the total number of hNPC plated was: 25% of neurons and 5% of glial cells with rat primary neurons, 6% of neurons and 5% of glial cells on matrigel in neuronal differentiating medium and 17% of neurons and 5% of glial cells on coculture with rat glial cells. These data suggest that the rate and the speed of differentiation of hNPCs were strictly dependent on the strategy used that has a greatest influence on neuronal differention than on glial differentiation. We also found that in absence of retinoic acid we obtained only glutamatergic excitatory neurons, while in presence of the drug during the differentiation protocols about 15% of the neurons obtained were inhibitory GABAergic neurons.

### Morphological and electrophysiological comparison between differentiation protocols

**Figure 7:**
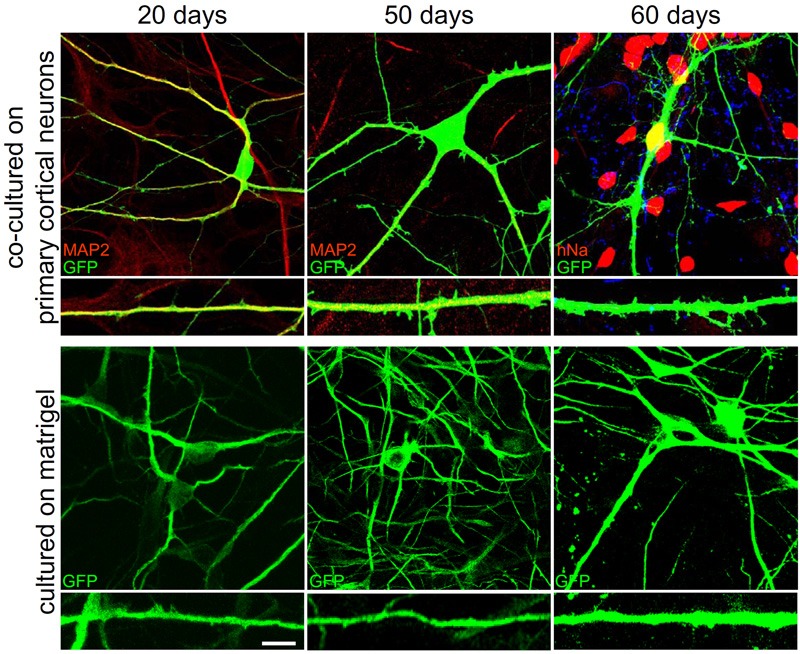
**Morphological comparison of neurons obtained with different differentiation protocols.** The panels show representative images of neurons and dendrites of hNP-derived neurons which have been differentiated by co-culture on E18 rat primary cortical neurons or on matrigel after 20, 50, and 60 days in culture.

We then compared the three developmental protocols considering dendrite development, formation of dendritic spines and synapses, and electrophysiological properties. At 20, 50, and 60 days we measured the number of dendrites and their mean length. Interestingly, we found that both number of dendrites and mean length were not significantly different between different ages of development and different differentiation protocols (Figure 7; Table 1). Only the dendritic mean length of hNP-derived neurons grown on matrigel after 20 days of culture are statistically smaller compare to hNP-derived neurons grown with the other protocols (Table 1). We then measured the number of dendritic spines, as a measure of synapse formation, during development. Opposite to dendritic development, we were not able to measure dendritic spines and synaptic protein clusters earlier than 50 days in culture, indicating that synapse maturation occurs later in already developed dendrites. Interestingly, when we compared the three different developmental protocols, we found that at the same culture age the hNP-derived neurons grown on rat primary cortical neurons had more dendritic spines and more synapses compared to the other two protocols (at 50 days in culture we measured a mean of 1.7 ± 0.4 spines per 10 μm on hNP-derived neurons grown on rat primary cortical neurons, a mean of 0.3 ± 0.1 spines per 10 μm on hNP-derived neurons grown on matrigel and a mean 0.4 ± 0.2 spines per 10 μm on hNP-derived neurons grown on glial cells; at 60 days in culture we measured a mean of 3.6 ± 1.1 spines per 10 μm on hNP-derived neurons grown on rat primary cortical neurons, a mean of 0.6 ± 0.3 spines per 10 μm on hNP-derived neurons grown on matrigel, and a mean 1.6 ± 0.4 spines per 10 μm on hNP-derived neurons grown on glial cells).

**Table 1:** **Quantitative comparison between differentiation protocols**.

	**20 days**	**50 days**	**60 days**
**NUMBER OF DENDRITES, MEAN LENGHT IN μm (n. of Cells Analyzed)**			
Coculture with rat primary neurons	3.2 ± 1.1	3.7 ± 1.1	3.2 ± 1.1
	82.2 ± 8.5	86.1 ± 7.4	78.4 ± 9.3
	(*n* = 24)	(*n* = 30)	(*n* = 21)
Coculture with rat primary neurons with RA	nm	3.3 ± 0.8	3.1 ± 1.4
		81.2 ± 8.5	84.4 ± 6.8
		(*n* = 27)	(*n* = 18)
Culture on matrigel in neuronal differentiating medium	3.8 ± 0.8	4.0 ± 0.7	4.3 ± 1.5
	52.3 ± 4.5	79.5 ± 4.6	83.8 ± 7.6
	^*^(*n* = 19)	(*n* = 19)	(*n* = 21)
Coculture with rat glial cells	nm	3.5 ± 0.8	2.8 ± 1.6
		90.6 ± 6.9	88.5 ± 9.8
		(*n* = 28)	(*n* = 26)
**NUMBER OF DENDRITIC SPINES PER 10 μm (n. of Cells Analyzed)**			
Coculture with rat primary neurons	0 (*n* = 14)	1.7 ± 0.4	3.6 ± 1.1
		(*n* = 10)	(*n* = 11)
Coculture with rat primary neurons with RA	nm	nm	2.9 ± 0.6
			(*n* = 8)
Culture on matrigel in neuronal differentiating medium	0 (*n* = 19)	0.3 ± 0.1	0.6 ± 0.3
		^**^(*n* = 12)	^**^(*n* = 14)
Coculture with rat glial cells	0 (*n* = 9)	0.4 ± 0.2	1.6 ± 0.4
		^**^(*n* = 10)	^**^(*n* = 10)
**% OF CELLS WITH APPRECIABLE SODIUM CURRENT (n. of Cells Analyzed)**			
Coculture with rat primary neurons	nm	100% (*n* = 11)	100% (*n* = 6)
Coculture with rat primary neurons with RA	nm	100% (*n* = 9)	nm
Culture on matrigel in neuronal differentiating medium	0% (*n* = 15)	21.4%	66.6%
		^**^(*n* = 14)	^**^(*n* = 12)
Coculture with rat glial cells	nm	nm	80%
			^**^(*n* = 10)

* *p < 0.05 compare to c. with rat primary neurons. nm, non measured*.

** *p < 0.01 compare to c. with rat primary neurons. nm, non measured*.

As mentioned before, recordings of total ionic currents showed that after 50–60 days in culture all of the terminal differentiation conditions were able to generate hNP-derived cells displaying voltage-gated sodium currents, which is a specific feature of mature neurons (Figures 3B, 4B, 5C, 6C; Table 1). Considering only the cells that displayed sodium currents, transient peak sodium inward currents had on average similar amplitude in the different conditions: maximal peak current density was 103 ± 13 pA/pF (*n* = 17) in cocultures with primary cortical neurons, 108 ± 20 pA/pF (*n* = 9) in cocultures with primary cortical neurons incubated with retinoic acid; 81 ± 21pA/pF (*n* = 8) in cocultures with glial cells, and 93.5 ± 13.2 pA/pF (*n* = 11) with differentiation medium (no statistically significant difference: Kruskall-Wallis test). However, the fraction of hNP-derived cells recorded after 50–60 days in culture which showed appreciable sodium current was different according to the differentiation protocol: 17/17 cells when co-cultured with rat primary neurons without retinoic acid, 9/9 when co-cultured with rat primary neurons in the presence of retinoic acid, 8/10 cells when co-cultured with glial cells, and 11/26 when they were grown in matrigel in differentiation medium (*p* < 0.01; Freeman-Halton extension of Fisher's exact test).

hNP-derived neurons were also able to produce fire discharges of action potentials in response to injection of depolarizing current steps; in order to compare them in the same condition, we maintained their resting potential at −70 mV injecting the appropriate holding current. All the cells displaying appreciable sodium current were excitable and generated overshooting action potentials (Figures 3C, 4C, 5D, 6D), which is a further specific feature of mature neurons.

Consistently with the morphological data, we recorded spontaneous synaptic activity at 50–60 days in hNP-derived neurons obtained with all the differentiation protocols, showing that they can form active networks. However, the frequencies of excitatory postsynaptic currents were higher in co-cultures with primary cortical rat neurons (average frequency in recordings of 125 s: 0.40 ± 0.13 Hz without retinoic acid, *n* = 15 cells; 0.23 ± 0.17 Hz without retinoic acid, *n* = 5 cells) compared to cells co-cultured with glia or grown on matrigel in differentiation medium (0.02 ± 0.01 Hz, *n* = 5 cells, 0.03 ± 0.01 Hz, *n* = 5 cells, respectively; *p* < 0.05 Kruskall-Wallis test).

**Figure 8:**
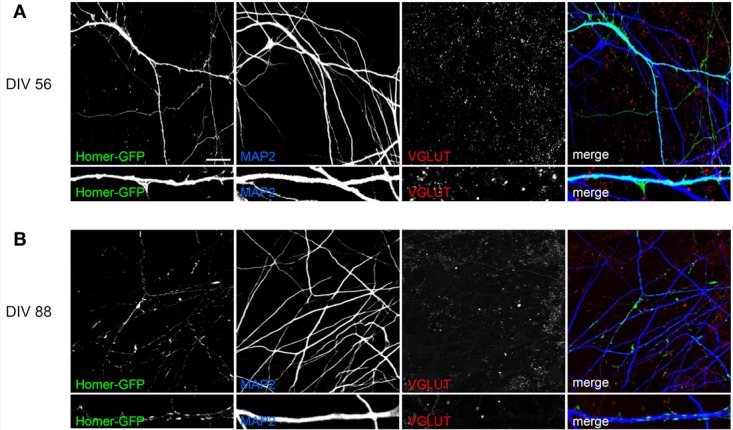
**Characterization of postsynapse formation in hNP-derived neurons differentiated by co-culture on E18 rat primary cortical neurons. (A)** After 56 days in co-culture, hNP-derived neurons are positive for MAP2 but the Homer-GFP signal is spread throughout the entire neuron. Scale bar, 50 μm. **(B)** After 88 days of co-culture, hNP-derived neurons are positive for MAP2 and the Homer-GFP signal is clustered with the classical synapse distribution. Scale bar, 15 μm.

To follow-up synapses formation during neuronal differentiation onto cortical neurons (the protocol that we considered the best for synapse formation), hNPs were infected with a lentivirus expressing Homer-GFP under CMV promoter. After 56 days of differentiation, the Homer-GFP signal was strong and diffused through the entire neuron indicating that, even in the presence of synapses and dendritic spines (Figure 7; Table 1), at this stage neurons are not sufficiently mature to cluster Homer at excitatory synapses. Only after 88 days in co-culture the Homer-GFP signal clustered with the classical synapses distribution (Figures 8A,B), suggesting that even if they are electrophysiologically active after 60 days in co-culture, these neurons need more time to develop more morphologically mature postsynapses.

## Discussion

Various studies have demonstrated the ability to generate neural stem cells (NSCs) from different sources such as hESCs, iPSCs or fibroblasts (Hochedlinger and Plath, 2009; Miura et al., 2009; Fong et al., 2010; Zhou et al., 2010; Kim et al., 2011; Peljto and Wichterle, 2011; Eiraku and Sasai, 2012; Han et al., 2012; Lujan et al., 2012; Sheng et al., 2012; Thier et al., 2012; Yamanaka, 2012).

Here we describe the establishment of replicating human neural-precursor cell lines, hNPs, from hiPSCs and their differentiation into mature neurons with different protocols, showing that they display different developmental properties *in vitro*, depending on the protocol used. The great difference between hiPSCs and hESCs is that, even they are both human stem cells, only hiPSCs can be collect from patients representing a unique model of pathology. For this reason we decided to analyse only neuronal differentiation of hNPC derived by hiPSC without considering hESC as control because the main aims of our project was to develop protocol suitable for hiPSCs.

Our hNP lines were obtained by inducing the neural phenotype through the generation of EBs and rosettes. hNPC were obtained by EBs formation instead of using dual SMAD inhibitors because we found that the EBs method is cheaper and more reproducible (Marchetto et al., 2010; Tang et al., 2013; Yuan et al., 2013). These hNPs did not express markers of pluripotency such as OCT 3/4 and Tra-1-81 and Nanog while expressing Nestin, an early neural lineage marker (Figures 2C–N) which was progressively lost following the induction of terminal differentiation (Figure 5A). The expression of Nestin, however, was maintained for up to 30 passages in all hNP cells, with only a minority of them being also positive for the glial marker GFAP, suggesting that the hNP lines are stable and do not spontaneously differentiate when grown in a medium containing bFGF and EGF (see Materials and Methods).

In all three *in vitro* protocols we used to differentiate hNPs we were able to obtain functional neurons that could be studied biochemically (when grown on matrigel), morphologically and electrophysiologically. Interestingly, the best neuronal maturation—measured as amount of dendritic spines, mature synapse, synaptic activity and fraction of recorded cells with appreciable sodium current—was obtained when we co-cultured the hNPs with primary rat cortical neurons. This might indicate that hNP-derived neurons can form synapses with rat neurons, or that the direct contact with rat neurons and/or the conditioned medium of the primary culture better support the differentiation of hNP cells. To note, we may exclude the latter possibility because we were not able to induce the same differentiation by simply using the primary culture conditioned medium (data not shown).

It's relevant to underline that the overall dendritic development was not differentially regulated by the different protocol used. As shown in Table 1, the number and mean length of dendrites was not statistically different between the different protocols used already after 20 days. Indeed our data suggest that dendritic development is already complete at 20 days, although these neurons are not electrophysiologically mature (Table 1). Thus, our results suggest that in these cultures neuronal dendrite formation occurs largely before the functional expression of sodium channels and the formation of synapses, and does not depend on the differentiation methods. Interestingly, during the synapse maturation period we didn't observe any further development of the dendritic arborization, suggesting that these two differentiation processes are mostly independent. Overall, our results show that the co-culture with primary cortical neurons is a condition that favors both electrophysiological maturation and synapse formation.

Although we observed synaptic activity after 50–60 days of culture, we also showed that, in order to obtain completely mature synaptic clustering of transfected GFP-Homer1b, more than 80 days of culture were required (Figure 8B). This data suggests that, similarly to what demonstrated for primary neuronal cultures (Bresler et al., 2004), the assembly of the postsynaptic density occurs temporally after the formation of the pre-synaptic compartment and of a functional electrophysiologically measurable synapse. Thus, our data suggests that the greatest difference between differentiation of hNPs and primary neurons *in vitro* is the time required for maturation: weeks for hNPs, days for primary neurons. Although the long period of time required for hNP neuronal differentiation is probably an intrinsic property, our data suggest that it's possible to accelerate this process by changing the culture environment, as in co-cultures with primary neurons.

Our data clearly suggest that the co-culture method is the most efficient to obtain mature neurons. In order to use hiPSCs as model for brain diseases is essential to differentiate them into functional neurons; our data clearly suggest that the co-culture method is the most efficient to obtain mature neurons both excitatory and inhibitory. It will be interesting to study if different type of primary neuron culture should be used to generate specific neurons subtype. The capability of our hNPs to differentiate in different subtypes of neurons is confirmed by the ability of retinoic acid to induce the maturation of inhibitory neurons (Addae et al., 2012). The differentiation potential of these cells into glial cells has been also determined (data not shown).

In summary, we describe a simple culture procedure to reproducibly and efficiently generate hNP cell lines from human iPSCs. These cells self-renew rapidly and can be stimulated to mature into different subtypes of neurons. We show that the rate of differentiation is strictly dependent on the strategy used to differentiate hNP cells and that the co-culture with rat cortical neurons is the most efficient way to obtain completely functional and morphologically developed neurons. The ability to obtain mature and functional neurons from self-renewing hNPs has major implications for regenerative medicine. Moreover, these lines will provide innovative experimental platforms to investigate mechanisms of neuronal differentiation and can serve as a model system for unveiling disease pathogenesis, for drug screening and toxicity tests.

### Conflict of interest statement

The authors declare that the research was conducted in the absence of any commercial or financial relationships that could be construed as a potential conflict of interest.
